# Ultrasound guidance practices used for the placement of vascular accesses in intensive care units: an observational multicentre study

**DOI:** 10.1186/s40001-023-01518-4

**Published:** 2023-11-16

**Authors:** Nathalie van der Mee-Marquet, Anne-Sophie Valentin, Isabelle Duflot, Mathilde Farizon, Agnès Petiteau, the collaboration group SPIADI, Alexandra Allaire, Elise Balestrat Sovic, Frédéric Barbut, Patrick Barthelemy, Pierre Berger, Marie-Camille Betti, Mathilde Blanié, Isabelle Cattaneo, Agnès Cecille, Hiba Chakaroun, Anne-Clémence Cholley, Marion David, Aude Davy, Joël Delhomme, Catherine Duval, Stéphanie Edouard, Laure Gabriele, Séverine Gallais, Colette Gerbier, Gilles Manquat, Valérie Goldstein, Florence Gourdon, Sylvie Joron, Anne-Marie Kayoulou-Bour, Gratienne Laethem, Florence Lemann, Martine Lemenager, Marie-Laure Lier, Malcie Mesnil, Nadine Mertel, Virginie Morange, Benoit Mottet, Stella Niot, Souad Ouzani, Christophe Perdrix, Amélie Renaud, Clémence Richaud, Catherine Rougier, Maryline Tarsac, Myriam Venelle, Isabelle Vidal

**Affiliations:** National Network for Surveillance and Prevention of Infections Associated with Invasive Devices (SPIADI Network), Centre d’Appui Pour la Prévention des Infections Associées Aux Soins (Cpias) Centre Val de Loire, Hôpital Bretonneau, Centre Hospitalier Régional Universitaire, 37044 Tours, France

**Keywords:** Intensive care unit, Ultrasound guidance, Infectious risk, Short-term central venous catheter, Peripheral inserted central catheter, Arterial catheter, Dialysis catheter, Long-lasting peripheral venous catheters, Improvement of practices

## Abstract

**Background:**

Central catheters expose ICU patients at risk of catheter-related bloodstream infections. A mechanism by which these infections occur is the contamination of the catheter during its insertion if aseptic techniques are not strictly applied. Recent studies suggest that the use of ultrasound guidance (USG) may increase the risk of catheter contamination during insertion. We assessed current practices regarding the use of USG during catheter insertion, with a focus on identifying breaches of the surgical asepsis required for this invasive procedure.

**Methods:**

In 26 intensive care units, we evaluated the use of USG during catheter insertion, using a questionnaire addressed to intensivists and direct observation of their practices.

**Results:**

We analyzed 111 questionnaires and 36 observations of intensivists placing catheters. The questionnaires revealed that 88% of intensivists used USG for catheter insertion. Among those using USG, 56% had received specific training, 17% benefited from specific recommendations, 76% marked the insertion site before skin antisepsis, and during catheter insertion, 96% used sterile gel and 100% used a sterile sheath and sterile gloves. We identified potential deviations from strict aseptic technique, including contact between the sheath and the needle (19.4%), handling of the US system during catheter insertion (2.8%), and use of sterile devices, where they were not yet necessary (during the marking site or skin antisepsis), resulting in their contamination at the time of catheter insertion.

**Conclusions:**

Interventions aimed at ensuring compliance with measures to prevent CRBs should be organized to prevent an increase in infections associated with US-guided catheter insertion.

**Supplementary Information:**

The online version contains supplementary material available at 10.1186/s40001-023-01518-4.

## Introduction

Critical care patients often require the use of intravascular catheters for their treatment, but these devices also increase the risk of catheter-related bloodstream infections (CRBs) [1]. One of the leading causes of CRBs is contamination during catheter insertion due to suboptimal skin antisepsis or failure to maintain aseptic conditions [2–4]. Ultrasound guidance (USG) is a recommended technique for catheter insertion as it allows for real-time visualization of the vessels during needle placement and travel, thereby improving accuracy and reducing complications [5–11]. However, CRBs have been reported due to the use of contaminated ultrasound gel [12, 13], and recent studies have associated the use of USG with an increased risk of CRBs [8]. However, the three studies described by Buetti et al., being retrospective and non-observational, the association between the use of USG for CVC placement and the increase in the incidence of CVC-related bacteremias could not be discussed in the context of all catheter insertion conditions. Although essential measures for optimal US-guided central venous catheter (CVC) insertion are well-established [10], the extent to which these measures are implemented in practice is unknown. To address this issue, we conducted an inventory of current practices regarding the use of USG during catheter insertion. We collected data through a questionnaire completed by intensivists responsible for device placement and conducted an observational study of their practices when inserting catheters using USG, with a specific focus on identifying breaches of the surgical asepsis required during catheter insertion.

## Methods

Between January 1st and July 31st, 2022, we conducted a two-part study among French intensivists. In the first part, we asked intensivists to describe their use of USG during the insertion of CVCs, dialysis catheters (DCs), arterial catheters (ACs), peripheral intravenous central catheters (PICClines) and MIDlines in ICU patients. The questionnaire collected information on the types of catheters inserted with USG, insertion sites, frequency of USG use, training, and the use of a procedure (Additional file 1). The questionnaires were distributed by the local infection control teams after providing information about the study. The second part of the study was an observational study. Local infection control teams observed intensivists placing catheters using USG using a standardized grid that included data on skin antisepsis, sterile gloving, type of gels used, use of a sterile protective sheath to cover the probe, and hand hygiene. We excluded emergency situations (Additional file 2). We analyzed the questionnaires and observation sheets at a national level, based on available recommendations [8–11] (Table 1).

**Table 1:** Specific expectations for intensivists using US guidance, practices evaluated and results obtained

Recommendations [10–13]	Evaluated criteria	Obtained results according to study method	
		Questionnaires (*n* = 111)	Observations (*n* = 36)
General recommendations			
The use of USG for the insertion of central venous catheter is recommended	HCWs using USG (systematically or frequently) for central venous catheter compared to the total *N* HCWs	98 (88.3%)	–
Training of HCWs in the use of USG is recommended	HCWs trained compared to N HCWs using USG	59 (56.2%)	16 (44.4%)
A procedure is available to HCWs who insert catheters using USG	HCWs having a procedure compared to *N* HCWs using USG	18 (17.1%)	–
Insertion site marking			
It is recommended to mark the insertion site using USG before skin antisepsis	HCWs carrying out marking before skin antisepsis (systematically or often) compared to *N* HCWs using USG	72 (75.6%)	21 (58.3)
A compliant hand rubbing is recommended before starting	HCWs carrying out compliant hand rubbing compared to *N* HCWs carrying out marking	–	10 (47.6%)
It is not recommended to cover the probe by a sterile sheath	HCWs not using a sheath compared to *N* HCWs carrying out marking	71 (72.4%)	15 (71.4%)
It is not recommended to use sterile gel	HCWs not using sterile gel compared to *N* HCWs carrying out marking	69 (70.4%)	16 (76.2%)
Skin antisepsis			
A compliant hand rubbing is recommended before starting	HCWs carrying out compliant hand rubbing compared to *N* HCWs carrying out antisepsis	–	17 (63.0%)
It is not recommended to glove	HCWs not gloving compared to *N* HCWs carrying out antisepsis	–	0
In case of gloving, it is recommended to remove gloves at the end of skin preparation	HCWs removing their gloves at the end of skin antisepsis compared to *N* HCWs gloving during this phase	–	15 (55.6%)
Insertion of the catheter			
A surgical hand disinfection followed by sterile gloving is recommended prior starting	HCWs carrying out compliant surgical hand disinfection and sterile gloving compared to the total *N* HCWs	–	12 (33.3%)
It is recommended to cover the probe and the connection cable of the US system with a sterile sheath when inserting the catheter			
	HCWs covering probe with a sterile sheath compared to the total *N* HCWs	105 (100.0%)	36 (100.0%)
	HCWs covering probe and cable with a sterile sheath compared to the total *N* HCWs	–	32 (88.9%)
It is recommended to use single-dose sterile gel applied inside and outside the sheath when inserting the catheter			
	HCWs using single-dose sterile gel compared to the total *N* HCWs	101 (96.2%)	36 (100.0%)
	HCWs applying the gel inside and outside the sheath compared to the total *N* HCWs	19 (18.1%)	5 (13.9%)
The tip of the needle must never come into contact with the sheath of the probe	Insertions with contact between the needle and the sheath related to the total *N* insertions	–	7 (19.4%)
The HCW must immediately change his gloves after handling the US system during catheter insertion	US system handling without glove changing related to the total *N* insertions	–	1 (2.8%)

## Results

A total of 26 ICUs located in distinct hospitals took part in the study.

### Questionnaire analysis

111 intensivists, including 26 residents (23.4%), completed the questionnaire (Table 1; Additional file 3: Tables S1 and S2). Among them, 94.6% reported using USG for catheter insertion, with the highest adoption rate for CVCs (93.7%), DCs (82.0%), and ACs (80.2%). USG use is systematic for CVCs and DCs while more variable for ACs. Of the 105 intensivists using USG, 56.2% received training on its use, as part of initial/continuing education (57.6%), or provided by a commercial company (22.0%) or by colleagues (20.3%); 17.1% of the intensivists benefited from specific recommendations for catheter insertion that take into account the use of USG. Before skin antisepsis, 93.3% of intensivists mark the insertion site using USG, with 27.6% using a sterile sheath to cover the probe and 29.6% using a sterile gel, although it is not necessary at this stage. During catheter insertion, all intensivists use a sterile sheath, 96.2% use single-dose sterile gel, and 17.9% report contact between the tip of the needle and the sheath, occurring systematically (*n* = 5), frequently (*n* = 2), sometimes (*n* = 10), or rarely (*n* = 5).

### Observational study (Tables 1)

36 intensivists, including 16 residents (44.4%), were observed inserting catheters [23 CVCs (63.9%), 7 MID lines (19.4%), 3 DCs (8.3%), 2 PICC lines (5.5%), and one AC (2.8%)]. Of the intensivists, 44.4% had received training on USG, mostly during their initial/continuing education (68.7%). Before skin antisepsis, 58.3% marked the insertion site using USG, with 28.6% using a sterile sheath and 23.8% using sterile gel. Compliance with skin antisepsis recommendations was satisfactory [i.e., skin cleaning (94.1%), application of alcoholic antiseptic solution (94.4%) with a sterile compress/applicator (100%), adherence to antiseptic drying time (100%)], except for the use of a 2% chlorhexidine alcoholic solution (36.1%) and hand hygiene compliance (63.0%). For catheter insertion, a sterile sheath covering the probe and cable was used in 88.9% of cases, and single-dose sterile gel was applied in all cases. However, only 33.3% of intensivists complied with surgical hand disinfection and/or wearing sterile gloves. In 19.4% of cases, the tip of the needle came into contact with the sheath, and one intensivist handled the US system without changing their gloves after the incident (2.8%) (Table 2).

**Table 2:** Distribution of the 36 intensivists according to hand hygiene (HH) and gloving compliance in the course of the catheter insertion, according to the occurrence of an insertion site marking and the participation of the operator to the skin antisepsis (each row corresponds to a specific observation sequence described in the successive columns

N HCWs	Insertion site marking		Participating in skin antisepsis				Insertion of the catheter		
	N HCWs	Compliant HH	N HCWs	Compliant HH	Gloving	Gloves' removal	Compliant HH	New sterile gloves	Adequate HH and gloving
8	1	1	8	8	8	8	8	8	8
1	1	0	1	1	1	1	1*	1	1
12	12	6	12	3	12	0	0	0	0
3	1	1	3	3	3	3	0	3	0
2	–	–	2	2	2	0	0	0	0
1	1	0	1	0	1	0	0	0	0
3	2	0	–	–	–	–	3	3	3
5	2	1	–	–	–	–	0	5	0
1	1	1	–	–	–	–	0	1	0
36	21	10	27	17	27	15	12	21	12

For example, in the first row, it involves 8 healthcare workers who perform hand hygiene and appropriate donning of sterile gloves (columns on the right side of the table) with the description of their practices from the site marking stage to the end of the antisepsis phase)

^*^One intensivist carried out double gloving during skin antisepsis and removed one pair of gloves before catheter insertion, what was considered acceptable practice

## Discussion

The use of USG has become a standard practice for CVC insertion [9]. However, the role of USG in the risk of CRBs is still debated. While it was not found to be a significant factor in some RCTs [14–17], a retrospective analysis of 3 RCTs found an association between USG and an increased risk of CRB (HR = 2.21, 95% CI 1.17–4.16) [8]. It is important to note that most randomized controlled trials did not specifically assess the infectious risk associated with USG [6–8]. Therefore, a thorough assessment of the infectious risk associated with the use of USG during CVC insertion is warranted.

Our study, conducted across 26 ICUs, utilized a questionnaire designed to elicit information on the intensivists’ use of USG during catheter insertion, and direct observation of intensivists inserting catheters. The data obtained from these two methods were concordant and complementary, shedding new light on the practice of US-guided catheter insertion. They first showed that USG is commonly used for inserting CVCs and DCs, and to a lesser extent for ACs, in line with current guidelines [9–11, 18]. However, only 50% of intensivists have received USG training, and most have not been given recommendations for catheter insertion that incorporate USG use. To promote best practices, regular USG training should be provided to intensivists, along with specific guidelines accessible to all ICUs [8]. Training should be conducted through simulation sessions that encompass both technical training for the use of ultrasound guidance to assist in catheter placement and the incorporation of hygiene rules to ensure strict asepsis during the procedure. To instill hygiene rules in the early stages of training for ultrasound guidance use, infection control specialists should be integrated into the teams responsible for scenario preparation. Our research has also identified potential deviations from strict aseptic technique. First, there are concerns related to the handling of the US system during catheter insertion and direct contact between the sheath and the catheter, as reported by intensivists and observed by infection control teams. Second, we have identified practices that do not ensure surgical aseptic conditions during insertion. In particular, among the intensivists who mark the insertion site, 33% use unnecessary sterile sheaths and single-dose sterile gel. This practice may result in the contamination of the sheath with the patient's skin flora, as skin antisepsis has not yet been performed, and if the sheath is not changed before catheter insertion, it can potentially contaminate the insertion site and the needle during catheter insertion. In addition, at the time of catheter insertion, intensivists follow the main recommendations to ensure a priori rigorous aseptic conditions (i.e., using a sterile sheath to protect the US probe, sterile gel, and sterile gloves), but for one-third of the intensivists, the sterile gloves are worn from the beginning of the skin antisepsis phase and are not changed before starting catheter insertion. Given these conditions, we suggest that the gloves, which are likely to be contaminated during the antisepsis phase, could serve as a source of catheter contamination during insertion. We propose raising awareness among intensivists about the infectious risks associated with the hasty use of sterile devices long before catheter insertion.

Our study has several limitations that should be acknowledged. First, our observations may not be fully representative of all intensivists. The study was conducted on a voluntary basis, and therefore, the participating hospitals and practitioners may have been particularly concerned with infectious risks, potentially leading to selection bias. We also cannot provide a response rate for our survey because we do not know the number of French intensivists performing central catheter insertions. Second, there may have been observation bias, particularly due to the Hawthorne effect. However, it should be noted that all French regions were represented, and the proportion of private and public hospitals was similar to that observed in France. Overall, these limitations are more likely to have minimized the extent of noncompliance with infection control policies than to have increased it.

## Conclusion

To prevent an increase in infections associated with USG during central catheter insertion, it is necessary to implement interventions aimed at ensuring compliance with measures to prevent catheter-related bloodstream infections, taking into account the specifics of catheter placement using USG.

### Supplementary Information

**Additional file 1:** Questionnaire for operators, aimed at describing their use of ultrasound guidance for catheter insertion.

**Additional file 2:** Standardized grid for observing the placement of central catheters.

**Additional file 3: Table S1.** US guidance use (%) by 111 intensivists for catheter insertion, according to catheter type. **Table S2**. Use of gel and sheath (%) by 105 HCWs for site marking and catheter insertion.

## Data Availability

The data sets used and/or analysed during the current study are available from the corresponding author N. van der Mee-Marquet on reasonable request.
