# Supplementary material for: Ultrasound guidance practices used for the placement of vascular accesses in intensive care units: an observational multicentre study
Source: Eur J Med Res. 2023 Nov 16;28:528. doi: 10.1186/s40001-023-01518-4 (PMC10652560; doi:10.1186/s40001-023-01518-4)
Supplement: Supplementary file 1 — Additional file 1: Questionnaire for operators, aimed at describing their use of ultrasound guidance for catheter insertion. [file 40001_2023_1518_MOESM1_ESM.pdf]

Questionnaire « Opérateurs » Échoguidage et pose des Cathéters Centraux et Midlines avec prolongateur intégré

FINESS ÉTABLISSEMENT	I__I__I__I__I__I__I__I__I__I					
NUMÉRO DE FICHE	I__I__I__I__I					
SERVICE	DISC I__I__I__I	SPE I__I__I__I	UF I__I__I__I__I__I__I__I			
**QUI ETES-VOUS ?**						
**VOTRE FONCTION**	**1. MÉDECIN**	**2. IADE**	**3. IDE**	**4. MANIP. RADIO**	**5. AUTRE => PRÉCISER**	**I__I**
**ÊTES-VOUS ÉTUDIANT?**				1. OUI	2. NON	**I__I**
**SI MÉDECIN,** VOTRE SPÉCIALITÉ :	**1. ANESTHÉSISTE**	**2. RÉANIMATEUR**	**3. ANESTHÉSISTE/RÉANIMATEUR**			**I__I**
	**4. RADIOLOGUE**	**5. URGENTISTE**	**6. AUTRE => PRÉCISER**			
**DISPOSEZ-VOUS D'UNE AIDE** LORS DE LA POSE DU CATHETER CENTRAL ?				1. OUI	2. NON	**I__I**
**SI IDE/IADE/MANIP. RADIO,** LA POSE DU CATHETER EST FAITE DANS LE CADRE D'UN PROTOCOLE DE COOPÉRATION AVEC UN MEDECIN ?				1. OUI	2. NON	**I__I**
**LA POSE DES CATHETERS CENTRAUX AVEC ECHOGUIDAGE**						
**UTILISEZ-VOUS L'ECHOGUIDAGE POUR LA POSE DE CATHETERS CENTRAUX ET MIDLINES AVEC PROLONGATEUR INTEGRE ?**				1. OUI	2. NON	**I__I**
**SI NON**	**MERCI (fin du questionnaire)**					
**SI OUI**	**POURSUIVEZ le remplissage du questionnaire ci-dessous**					
**QUELS CATHÉTERS POSEZ-VOUS AVEC ECHOGUIDAGE ?**	**1. CVC**			1. OUI	2. NON	**I__I**
	**SI OUI 1. SYSTEMATIQUEMENT**	**2. SOUVENT**	**3. PARFOIS**	**4. RAREMENT**		**I__I**
	**2. CATHÉTER ARTÉRIEL**			1. OUI	2. NON	**I__I**
	**SI OUI 1. SYSTEMATIQUEMENT**	**2. SOUVENT**	**3. PARFOIS**	**4. RAREMENT**		**I__I**
	**3. PICCline**			1. OUI	2. NON	**I__I**
	**SI OUI 1. SYSTEMATIQUEMENT**	**2. SOUVENT**	**3. PARFOIS**	**4. RAREMENT**		**I__I**
	**4. MIDline AVEC PROLONGATEUR INTÉGRÉ**			1. OUI	2. NON	**I__I**
	**SI OUI 1. SYSTEMATIQUEMENT**	**2. SOUVENT**	**3. PARFOIS**	**4. RAREMENT**		**I__I**
	**5. CATHÉTER DE DIALYSE**			1. OUI	2. NON	**I__I**
	**SI OUI 1. SYSTEMATIQUEMENT**	**2. SOUVENT**	**3. PARFOIS**	**4. RAREMENT**		**I__I**
	**6. AUTRE => PRÉCISER**			1. OUI	2. NON	**I__I**
**EN MOYENNE PAR MOIS, COMBIEN DE CATHÉTERS (tous types) POSEZ-VOUS AVEC ECHOGUIDAGE ?**						**I__I__I**
**QUELS SONT LES LIEUX OU VOUS POSEZ DES CATHETERS AVEC ECHOGUIDAGE ?**	**1. BLOC OP.**			1. OUI	2. NON	**I__I**
	**SI OUI 1. SOUVENT**	**2. PARFOIS**	**3. RAREMENT**	**4. JAMAIS**		
	**2. RADIO. INTERV.**			1. OUI	2. NON	**I__I**
	**SI OUI 1. SOUVENT**	**2. PARFOIS**	**3. RAREMENT**	**4. JAMAIS**		
	**3. MEDECINE**			1. OUI	2. NON	**I__I**
	**SI OUI 1. SOUVENT**	**2. PARFOIS**	**3. RAREMENT**	**4. JAMAIS**		
	**4. REANIMATION**			1. OUI	2. NON	**I__I**
	**SI OUI 1. SOUVENT**	**2. PARFOIS**	**3. RAREMENT**	**4. JAMAIS**		
	**5. SOINS INTENSIFS**			1. OUI	2. NON	**I__I**
	**SI OUI 1. SOUVENT**	**2. PARFOIS**	**3. RAREMENT**	**4. JAMAIS**		
	**6. URGENCES**			1. OUI	2. NON	**I__I**
	**SI OUI 1. SOUVENT**	**2. PARFOIS**	**3. RAREMENT**	**4. JAMAIS**		
	**7. AUTRE=> PRÉCISER**			1. OUI	2. NON	**I__I**
**VOTRE FORMATION À LA POSE D'UN CATHÉTER AVEC ECHOGUIDAGE**						
**AVEZ-VOUS RECU UNE FORMATION A l'ÉCHOGUIDAGE?**				1. OUI	2. NON	**I__I**
**SI OUI**	**DATE** DE LA FORMATION (la plus récente) A L'ECHOGUIDAGE			1. ≤1 an	2. > 1 an	**I__I**
	**TYPE** DE FORMATION	1. DIU	2. SOCIETE	3. AUTRE => PRÉCISER		**I__I**
**LA POSE D'UN CATHÉTER AVEC ECHOGUIDAGE ET VOTRE SERVICE**						
**AVEZ-VOUS A DISPOSITION UNE PROCÉDURE INSTITUTIONNELLE?**			**1. OUI**	**2. NON**	**3. NSP**	**I__I**
**SI OUI**	**LA PROCÉDURE EST VALIDEE PAR LE CLIN (ou structure apparentée)**		**1. OUI**	**2. NON**	**3. NSP**	**I__I**
**ELLE MENTIONNE**	**LES INDICATIONS DE L'ÉCHOGUIDAGE**		**1. OUI**	**2. NON**	**3. NSP**	**I__I**
	**LE TYPE DE GAINE / HOUSSE A UTILISER**		**1. OUI**	**2. NON**	**3. NSP**	**I__I**
	**LE TYPE DE GEL POUR LE REPERAGE**		**1. OUI**	**2. NON**	**3. NSP**	**I__I**
	**LE TYPE DE GEL POUR LA POSE DU CATHETER**		**1. OUI**	**2. NON**	**3. NSP**	**I__I**
	**LES MODALITÉS D'ENTRETIEN DE L'APPAREIL**		**1. OUI**	**2. NON**	**3. NSP**	**I__I**
	**LA TRACABILITE DE L'ENTRETIEN DE L'APPAREIL**		**1. OUI**	**2. NON**	**3. NSP**	**I__I**
	**LA TRACABILITE DE L'ENTRETIEN DE LA SONDE**		**1. OUI**	**2. NON**	**3. NSP**	**I__I**

**LE REPÉRAGE AVANT LA PREPARATION CUTANEE (ANTISEPSIE)**					
**REALISEZ –VOUS UN REPERAGE AVANT L'APPLICATION DE L'ANTISEPTIQUE**			**1. OUI**	**2. NON**	**I__I**
**SI OUI**	**1. SYSTEMATIQUEMENT 2. SOUVENT 3. PARFOIS 4. RAREMENT**				**I__I**
**SI OUI**	**LE GEL UTILISE EST :**	**1. MONODOSE STERILE 2. MONODOSE NON STERILE 3. FLACON**			**I__I**
**SI OUI**	**UTILISEZ-VOUS UNE GAINE / HOUSSE?**	**1. STERILE 2. NON STERILE 3. NON**			**I__I**
**L'INSERTION DU CATHETER SOUS ECHOGUIDAGE**					
**ATTENDEZ-VOUS LE SÉCHAGE SPONTANÉ DE L'ANTISEPTIQUE AVANT D'APPLIQUER LE GEL?**			**1. OUI**	**2. NON**	**I__I**
**SI OUI**	**1. SYSTEMATIQUEMENT 2. SOUVENT 3. PARFOIS 4. RAREMENT**				I__I
**UTILISEZ-VOUS UNE GAINE / HOUSSE ?**			**1. OUI**	**2. NON**	**I__I**
**SI OUI**	**1. SYSTEMATIQUEMENT 2. SOUVENT 3. PARFOIS 4. RAREMENT**				I__I
**SI OUI**	**LA GAINE / HOUSSE EST-ELLE STERILE ?**		**1. OUI**	**2. NON**	**I__I**
**SI OUI**	**LA GAINE / HOUSSE RECOUVRE-T-ELLE LA SONDE ET LE CABLE DE L'APPAREIL ?**		**1. OUI**	**2. NON**	**I__I**
**SI OUI**	**LA GAINE / HOUSSE EST-ELLE SPECIFIQUE POUR L'ECHOGUIDAGE ?**		**1. OUI**	**2. NON**	**I__I**
**LE GEL UTILISE EST :**	**1. MONODOSE STERILE 2. MONODOSE NON STERILE 3. FLACON**				I__I
**APPLIQUEZ –VOUS LE GEL 1. SUR LA GAINE 2. SUR LA SONDE 3. SUR LA GAINE ET LA SONDE**					I__I
**LA SONDE ENTRE-T-ELLE EN CONTACT AVEC L'AIGUILLE DE PONCTION ?**			**1. OUI**	**2. NON**	**I__I**
**SI OUI**	**1. SYSTEMATIQUEMENT 2. SOUVENT 3. PARFOIS 4. RAREMENT**				I__I
**A LA FIN DE L'INSERTION DU CATHETER SOUS ECHOGUIDAGE**					
**POSEZ-VOUS LA SONDE SUR LE SUPPORT PREVU AU NIVEAU DE L'ECHOGRAPHE ?**			**1. OUI**	**2. NON**	**I__I**
**INSPECTEZ-VOUS LA GAINE / HOUSSE ?**			**1. OUI**	**2. NON**	**I__I**
**SI OUI**	**1. SYSTEMATIQUEMENT 2. SOUVENT 3. PARFOIS 4. RAREMENT**				**I__I**
**INSPECTEZ-VOUS LA SONDE ?**			**1. OUI**	**2. NON**	**I__I**
**SI OUI**	**1. SYSTEMATIQUEMENT 2. SOUVENT 3. PARFOIS 4. RAREMENT**				**I__I**
**SI OUI**	**1. A L'OEIL NU 2. PAR ESSUYAGE AVEC COMPRESSE**				**I__I**
**ENTRETIEN DE LA SONDE ET DE L'ÉCHOGRAPHE**					
**UN ESSUYAGE HUMIDE DE LA SONDE D'ÉCHOGRAPHIE EST-IL REALISE JUSTE AVANT QUE VOUS UTILISIEZ L'ECHOGRAPHE ?**		1. OUI	2. NON	3. NSP	I__I
SI OUI	1. SYSTEMATIQUEMENT 2. SOUVENT 3. PARFOIS 4. RAREMENT				I__I
SI OUI	1. PAR L'AIDE 2. PAR VOUS			3. NSP	I__I
SI OUI	LE PRODUIT UTILISÉ EST UN 1. DETERGENT 2. DÉTERGENT/DÉSINFECTANT			3. NSP	I__I
**UN ESSUYAGE HUMIDE DE LA SONDE D'ÉCHOGRAPHIE EST-IL REALISE A LA FIN DU REPERAGE?**		**1. OUI**	**2. NON**	**3. NSP**	**I__I**
SI OUI	1. SYSTEMATIQUEMENT 2. SOUVENT 3. PARFOIS 4. RAREMENT				I__I
SI OUI	1. PAR L'AIDE 2. PAR VOUS			3. NSP	I__I
SI OUI	LE PRODUIT UTILISÉ EST UN 1. DETERGENT 2. DÉTERGENT/DÉSINFECTANT			3. NSP	I__I
**UN ESSUYAGE HUMIDE DE LA SONDE D'ÉCHOGRAPHIE EST-IL REALISE JUSTE AVANT L'INSERTION?**		**1. OUI**	**2. NON**	**3. NSP**	**I__I**
SI OUI	1. SYSTEMATIQUEMENT 2. SOUVENT 3. PARFOIS 4. RAREMENT				I__I
SI OUI	1. PAR L'AIDE 2. PAR VOUS			3. NSP	I__I
SI OUI	LE PRODUIT UTILISÉ EST UN 1. DETERGENT 2. DÉTERGENT/DÉSINFECTANT			**3. NSP**	**I__I**
**A LA FIN DE L'INSERTION, SI UNE DECHIRURE DE LA GAINE OU DES SALISSSURES DE LA SONDE SONT OBSERVEES :**					
UN NETTOYAGE ET UNE DESINFECTION DE LA SONDE SONT-ILS REALISES ?		1. OUI	2. NON	3. NSP	I__I
SI OUI	1. SYSTEMATIQUEMENT 2. SOUVENT 3. PARFOIS 4. RAREMENT				I__I
SI OUI	1. PAR L'AIDE 2. PAR VOUS			3. NSP	I__I
SI OUI	LE PRODUIT UTILISÉ EST UN 1. DETERGENT 2. DÉTERGENT/DÉSINFECTANT			3. NSP	I__I
SI OUI	1. NETTOYAGE HUMIDE AVEC DETERGENT/DESINFECTANT 2. PAR IMMERSION 3. NSP				I__I
**A LA FIN DE L'INSERTION, SI LA GAINE / HOUSSE EST INTACTE OU VOUS N'OBSERVEZ AUCUNE SALISSSURE**					
UN NETTOYAGE ET UNE DESINFECTION DE LA SONDE SONT-ILS REALISES ?		1. OUI	2. NON	3. NSP	I__I
SI OUI	1. SYSTEMATIQUEMENT 2. SOUVENT 3. PARFOIS 4. RAREMENT				I__I
SI OUI	1. PAR L'AIDE 2. PAR VOUS			3. NSP	I__I
SI OUI	LE PRODUIT UTILISÉ EST UN 1. DETERGENT 2. DÉTERGENT/DÉSINFECTANT			3. NSP	I__I
SI OUI	1. NETTOYAGE HUMIDE AVEC DETERGENT/DESINFECTANT 2. PAR IMMERSION 3. NSP				I__I
**APRES LA POSE, UN ESSUYAGE HUMIDE DE L'ÉCHOGRAPHE EST RÉALISÉ?**		**1. OUI**	**2. NON**	**3. NSP**	**I__I**
SI OUI	1. SYSTEMATIQUEMENT 2. SOUVENT 3. PARFOIS 4. RAREMENT				I__I
SI OUI	1. PAR L'AIDE 2. PAR VOUS			3. NSP	I__I
SI OUI	LE PRODUIT UTILISÉ EST UN 1. DETERGENT 2. DÉTERGENT/DÉSINFECTANT			3. NSP	I__I
SI OUI	IL COMPREND : L'ECRAN	1. OUI	2. NON	3. NSP	I__I
SI OUI	IL COMPREND : LE CLAVIER	1. OUI	2. NON	3. NSP	I__I
SI OUI	IL COMPREND : LE CABLE	1. OUI	2. NON	3. NSP	I__I
**APRES LA POSE, L'ÉCHOGRAPHE EST STOCKÉ A L'ABRI DES CONTAMINATIONS**		**1. OUI**	**2. NON**	**3. NSP**	**I__I**

MERCI !
