# Supplementary material for: Ultrasound guidance practices used for the placement of vascular accesses in intensive care units: an observational multicentre study
Source: Eur J Med Res. 2023 Nov 16;28:528. doi: 10.1186/s40001-023-01518-4 (PMC10652560; doi:10.1186/s40001-023-01518-4)
Supplement: Supplementary file 2 — Additional file 2: Standardized grid for observing the placement of central catheters. [file 40001_2023_1518_MOESM2_ESM.pdf]

FICHE ECHOGUIDAGEpour la pose des Cathéters Centraux et Midlines avec prolongateur intégré

FINESS ÉTABLISSEMENT				I__I__I__I__I__I__I__I__I__I
NUMÉRO DE FICHE P1				I__I__I__I__I
SITE D'INSERTION	1. JUGULAIRE	2. FÉMORAL	3. SOUS CLAVIER	I__I
	4. RADIAL	5. AUTRE => PRÉCISER		
L'OPERATEUR				
VOTRE FONCTION	1. MÉDECIN 2. IADE 3. IDE 4. MANIP. RADIO	5. AUTRE => PRÉCISER		I__I
ÉTUDIANT?		1. OUI	2. NON	I__I
L'AUDITE A RECU UNE FORMATION A l'ÉCHOGUIDAGE?		1. OUI	2. NON	I__I
SI OUI	DATE DE LA FORMATION (la plus récente) A L'ECHOGUIDAGE	1. ≤1 an	2. > 1 an	I__I
	TYPE DE FORMATION 1. DIU 2. SOCIETE	3. AUTRE => PRÉCISER		I__I
PREPARATION DE L'ECHOGRAPHE				
PREPARATION DE L'ECHOGRAPHE		1. OUI	2. NON	I__I
SI OUI	PREPARATION DE L'ECHOGRAPHE PAR	1. OPERATEUR	2. AIDE	I__I
	HYGIÈNE DES MAINS AVANT LA PREPARATION DE L'ECHOGRAPHE	1. OUI	2. NON	I__I
	SI OUI, 1. PAR FRICTION 2. LAVAGE SIMPLE			I__I
	SI OUI, LA TECHNIQUE EST-ELLE CONFORME?	1. OUI	2. NON	I__I
L'AUDITE REALISE UN ESSUYAGE HUMIDE DE LA SONDE D'ÉCHOGRAPHIE		1. OUI	2. NON	I__I
SI OUI	LE PRODUIT UTILISÉ : 1. DÉTERGENT 2. DÉTERGENT/DÉSINFECTANT 3. AUTRE (préciser)			I__I
LE REPÉRAGE AVANT LA PREPARATION CUTANEE (ANTISEPSIE)				
L'AUDITE FAIT UN REPERAGE AVANT L'APPLICATION DE L'ANTISEPTIQUE ?		1. OUI	2. NON	I__I
SI NON	ALLER A LA SECTION « PREPARATION CUTANEE »			
SI OUI	HYGIÈNE DES MAINS AVANT LE REPERAGE	1. OUI	2. NON	I__I
	Si OUI 1. PAR FRICTION 2. LAVAGE SIMPLE			I__I
	LA TECHNIQUE EST-ELLE CONFORME?	1. OUI	2. NON	I__I
	PORT DE GANTS POUR LE REPERAGE	1. OUI	2. NON	I__I
	SI OUI 1. GANTS STERILES 2. GANTS NON STERILES			I__I
	LE GEL UTILISE EST : 1. MONODOSE STERILE 2. MONODOSE NON STERILE 3. FLACON			I__I
	SI FLACON, LE VOLUME DU FLACON ? 1.≤250 mL 2.>250 mL			I__I
	LA DATE D'OUVERTURE EST NOTEE SUR LE FLACON ?	1. OUI	2. NON	I__I
	LE FLACON EST OUVERT DEPUIS : 1. ≤24 H 2. > 24 H 9. NSP			I__I
	LE FLACON TOUCHE PEAU OU SONDE PENDANT LE REPERAGE ?	1. OUI	2. NON	I__I
	LE FLACON EST RECAPUCHONNE APRES UTILISATION ?	1. OUI	2. NON	I__I
	LE GEL EST CHAUFFE AVANT UTILISATION ?	1. OUI	2. NON	I__I
	L'AUDITE UTILISE UNE GAINE / HOUSSE : 1. STERILE 2. NON STERILE 3. NON			I__I
	L'AUDITE REALISE UN ESSUYAGE HUMIDE DE LA SONDE A LA FIN DU REPERAGE?	1. OUI	2. NON	I__I
	SI OUI, LE PRODUIT UTILISÉ EST UN 1. DETERGENT 2. DÉTERGENT/DÉSINFECTANT 3. AUTRE (préciser)			I__I
	L'AUDITE POSE LA SONDE SUR SUPPORT PREVU AU NIVEAU DE L'ECHOGRAPHE A LA FIN DU REPERAGE	1. OUI	2. NON	I__I
LA PREPARATION CUTANEE (ANTISEPSIE)				
L'AUDITE FAIT LA PREPARATION CUTANEE ?		1. OUI	2. NON	I__I
SI NON	ALLER A LA SECTION « INSERTION DU CATHETER »			
L'AUDITE A REALISE UNE HYGIÈNE DES MAINS JUSTE AVANT DE DEBUTER L'ANTISEPSIE ?		1. OUI	2. NON	I__I
SI OUI,	1. PAR DCMF 2. PAR LAVAGE CHIRURG. 3. PAR FRICTION 4. LAVAGE SIMPLE			I__I
	LA TECHNIQUE EST-ELLE CONFORME?	1. OUI	2. NON	I__I
L'AUDITE ENFILE DES GANTS AVANT L'ANTISEPSIE ?		1. OUI	2. NON	I__I
SI OUI,	1. STERILES 2. NON STERILES			I__I
	DOUBLE GANTAGE ?	1. OUI	2. NON	I__I
	L'AUDITE RETIRE SES GANTS (OU 1 PAIRE SI DOUBLE GANTAGE) APRES L'ANTISEPSIE?	1. OUI	2. NON	I__I

L'INSERTION DU CATHETER					
L'AUDITE REALISE UNE HYGIÈNE DES MAINS JUSTE AVANT L'ENFILAGE DES GANTS ?		1. OUI	2. NON	3. NA	I__I
	SI OUI, 1. PAR DCMF 2. PAR LAVAGE CHIRURG. 3. PAR FRICTION 4. LAVAGE SIMPLE				I__I
	LA TECHNIQUE EST-ELLE CONFORME?	1. OUI	2. NON		I__I
L'AUDITE ENFILE DES GANTS :	1. STERILES 2. NON STERILES 3.NA				I__I
L'AUDITE UTILISE UNE GAINE / HOUSSE ?		1. OUI	2. NON		I__I
SI OUI	LA GAINE / HOUSSE EST-ELLE STERILE ?	1. OUI	2. NON		I__I
	LA GAINE / HOUSSE A-T-ELLE UN MARQUAGE CE ?	1. OUI	2. NON		I__I
	LA GAINE / HOUSSE EST-ELLE SPECIFIQUE POUR L'ECHOGUIDAGE ?	1. OUI	2. NON		I__I
	LA GAINE / HOUSSE RECOUVRE-T-ELLE LA SONDE ET LE CABLE DE L'APPAREIL ?	1. OUI	2. NON		I__I
LE SÉCHAGE SPONTANÉ DE L'ANTISEPTIQUE AVANT D'APPLIQUER LE GEL EST RESPECTE?		1. OUI	2. NON		I__I
LE GEL UTILISE EST : 1. MONODOSE STERILE 2. MONODOSE NON STERILE 3. NON STERILE EN FLACON (★Q1)					I__I
LE GEL EST APPLIQUE 1. SUR LA GAINE / HOUSSE 2. SUR LA SONDE 3. SUR LA GAINE ET LA SONDE					I__I
LA SONDE ENTRE EN CONTACT AVEC L'AIGUILLE DE PONCTION ?		1. OUI	2. NON		I__I
L'AUDITE MANIPULE L'ECHOGRAPHE PENDANT L'INSERTION DU CATHETER		1. OUI	2. NON		I__I
SI OUI	1. L'ECHOGRAPHE EST TOUCHE DIRECTEMENT AVEC LES GANTS STERILES (★Q2)				I__I
	2. L'ECHOGRAPHE EST TOUCHE EN UTILISANT DES COMPRESSES STERILES				
	3. L'ECHOGRAPHE EST TOUCHE EN UTILISANT DES COMPRESSES NON STERILES				
	4. L'ECHOGRAPHE EST TOUCHE AVEC UNE AUTRE METHODE (préciser)				
SI L'ECHOGRAPHE EST TOUCHE DIRECTEMENT AVEC LES GANTS STERILES	SI UNE PAIRE DE GANTS, LES GANTS SONT CHANGES IMMEDIATEMENT AVANT DE CONTINUER LA POSE ?	1. OUI	2. NON		I__I
	SI OUI, UNE HYGIÈNE DES MAINS EST FAITE AVANT D'ENFILER DE NOUVEAUX GANTS ?	1. OUI	2. NON		I__I
	SI OUI, PAR 1. DCMF 2. LAVAGE CHIRURG. 3. FRICTION 4. LAVAGE SIMPLE				I__I
	LA TECHNIQUE EST-ELLE CONFORME?	1. OUI	2. NON		I__I
	SI DOUBLE GANTAGE, 1 PAIRE DE GANTS EST IMMEDIATEMENT RETIREE	1. OUI	2. NON		I__I
A LA FIN DE LA POSE DU CATHETER					
GESTION DE LA SONDE					
LA GAINE / HOUSSE EST INSPECTEE A L'ŒIL NU		1. OUI	2. NON		I__I
LA SONDE EST INSPECTEE: 1. A L'ŒIL NU 2. PAR ESSUYAGE AVEC COMPRESSE 3. NON 4. NA					I__I
SI INSPECTION,	LA GAINE / HOUSSE PRESENTE UNE DECHIRURE ?	1. OUI	2. NON		I__I
	LA SONDE PRESENTE DES SALISSURES ?	1. OUI	2. NON		I__I
LA SONDE EST ELLE ENTRETENUE ?		1. OUI	2. NON		I__I
SI OUI, 1. ESSUYAGE HUMIDE AVEC DETERGENT/DESINFECTANT 2. PAR IMMERSION 3. AUTRE (préciser)					I__I
LA SONDE EST-ELLE DEPOSEE SUR SUPPORT PREVU AU NIVEAU DE L'ECHOGRAPHE		1. OUI	2. NON		I__I
GESTION DE L'ÉCHOGRAPHE					
L'ECHOGRAPHE BENEFICIE-T-IL D'UN ESSUYAGE HUMIDE ?		1. OUI	2. NON	3. NSP	I__I
SI OUI, IL COMPREND	LE CABLE	1. OUI	2. NON	3. NSP	I__I
	LE CLAVIER	1. OUI	2. NON	3. NSP	I__I
	L'ÉCRAN	1. OUI	2. NON	3. NSP	I__I
	LE PRODUIT UTILISÉ EST UN : 1. DÉTERGENT 2.DÉTERGENT/DÉSINFECTANT 3. AUTRE (préciser)				I__I
	L' ESSUYAGE EST REALISE PAR 1. OPERATEUR 2. AIDE 3. AUTRE (préciser)				I__I
ENTRETIEN AVEC L'AUDITE					
Q1	Pourquoi l'opérateur utilise un gel non stérile pour l'échoguidage lors de l'insertion du cathéter ?				I__I
	1. HABITUDE	2. RECO. MECONNUE	3. PRATIQUE DU SERVICE		
	4. PAS D'AUTRE CONDITIONNEMENT	5. AUTRE => PRÉCISER			
Q2	Pendant l'insertion du cathéter, pourquoi l'opérateur a poursuivi l'insertion du cathéter avec gants ayant touché le clavier de l'échographe ?				I__I
	1. HABITUDE	2. PEU DE RISQUE	3. AUTRE => PRÉCISER		
