# Supplementary material for: Ultrasound guidance practices used for the placement of vascular accesses in intensive care units: an observational multicentre study
Source: Eur J Med Res. 2023 Nov 16;28:528. doi: 10.1186/s40001-023-01518-4 (PMC10652560; doi:10.1186/s40001-023-01518-4)
Supplement: Supplementary file 3 — Additional file 3: Table S1. US guidance use (%) by 111 intensivists for catheter insertion, according to catheter type. Table S2. Use of gel and sheath (%) by 105 HCWs for site marking and catheter insertion. [file 40001_2023_1518_MOESM3_ESM.docx]

**Table S1**. US guidance use (%) by 111 intensivists for catheter insertion, according to catheter type.

| For | Number of HCWs using US guidance for catheter insertion | | | | |
| --- | --- | --- | --- | --- | --- |
|  | All | Practice frequency | | | |
|  |  | systematically | frequently | sometimes | rarely |
| At least one catheter type | 105 (94.6) |  |  |  |  |
| Short-term CVCs | 104 (93.7) | 77 (74.0) | 21 (20.2) | 6 (5.8) | 0 |
| Short-term dialysis catheter | 91 (82.0) | 72 (79.1) | 15 (16.5) | 4 (4.4) | 0 |
| Arterial catheters | 89 (80.2) | 14 (15.7) | 25 (28.1) | 31 (34.8) | 19 (21.3) |
| MID lines | 54 (48.6) | 50 (92.6) | 1 (1.8) | 3 (5.6) | 0 |
| PICC lines | 42 (37.8) | 38 (90.5) | 2 (4.8) | 2 (4.8) | 0 |

**Table S2**. Use of gel and sheath (%) by 105 HCWs for site marking and catheter insertion.

| Expected practices | Number of HCWs carrying out the expected practice | | | | |
| --- | --- | --- | --- | --- | --- |
|  | All | Practice frequency | | | |
|  |  | systematically | frequently | sometimes | rarely |
| Insertion site marking | 90 (85.7) | 66 (73.3) | 18 (20.0) | 6 (6.7) | 0 |
| with no sheath | 71 (67.6) |  |  |  |  |
| with non-sterile gel | 69 (65.7) |  |  |  |  |
| Catheter insertion | | | | | |
| with a sterile sheath | 105 (100.0) | 103 (98.1) | 2 (1.9) |  |  |
| with single-dose sterile gel | 101 (96.2) |  |  |  |  |
| with gel (inside and outside the sheath) | 19 (18.1) |  |  |  |  |
